# Correspondence

**Published:** 1902-03-15

**Authors:** 


### CORRESPONDENCE.

*Editor Dental Register:*—Dear Sir, two weeks ago I placed arsenical paste in a distal cavity of the upper first molar. After three days I found the paste had leaked out of the cavity, and the gum as far forward as the cuspid was badly congested. I applied tincture of aconite and iodine to the gums several times, massaging them each time. The swelling subsided without sloughing. I removed the pulp and filled the tooth which had become quite loose and somewhat sore. The gums look normal but bleed easily. The patient stated yesterday that the tooth gave her a headache. I gave the patient a mouth-wash of tannin, myrrh and arnica. Can you advise me of further or better treatment?—*E. S. U.*

The treatment will doubtless prevent suppuration and necrosis, the tendency in such cases. We doubt the advisability of permanently filling the roots of the tooth until all irritation has subsided. We would suggest that morning and evening the mouth should be disinfected before brushing the teeth with hydrogen peroxide, and that the teeth be kept scrupulously clean with a good antiseptic tooth powder, and that a stimulating mouth-wash of myrrh, calendula, or hydronaphthol be used three or four times each day for the next two weeks, and then at gradually lengthened periods. Gentle massaging and use of the tooth will also be helpful.—Ed.

---

*Editor Dental Register:*—Dear Sir, a boy sixteen years of age came to me for professional services recently and I was surprised to find that his teeth although perfectly developed had a very decided pink color. The six anterior teeth show the color more than the bicuspids and molars, especially near the incisal edges. The pink color shades to a straw color at the gum border. The patient says his desiduous teeth were perfect in color, but the permanent teeth have always been colored. His brothers and sisters are all free from the discoloration, but a cousin on the father's side shows the same peculiarity except that it is not so marked. Can anything be done in the way of bleaching such teeth? It is evidently a structural stain or pigment and not accidental.—*F. G. Swartz.*

We have never seen such a stain and do not think it at all probable that it could be removed without peril to the teeth. If any of our readers can throw any light on this case we shall be pleased to hear from them.—Ed.

